# *PLA2G6* Silencing Suppresses Melanoma Progression and Affects Ferroptosis Revealed by Quantitative Proteomics

**DOI:** 10.3389/fonc.2022.819235

**Published:** 2022-03-07

**Authors:** Yifei Wang, Hao Song, Qiuju Miao, Yan Wang, Jinliang Qi, Xiulian Xu, Jianfang Sun

**Affiliations:** ^1^Institute of Dermatology, Chinese Academy of Medical Sciences and Peking Union Medical College, Nanjing, China; ^2^State Key Laboratory of Pharmaceutical Biotechnology, School of Life Sciences, Nanjing University, Nanjing, China

**Keywords:** cutaneous malignant melanoma, pathogenesis, iPLA_2_β, ferroptosis, LC-MS/MS

## Abstract

Although phospholipase A2 group VI (*PLA2G6*) is involved in oncogenesis in several human tumors, its expression and role in cutaneous malignant melanoma (CMM) pathogenesis remains unclear. Here, by using the Oncomine and CCLE online database, immunohistochemistry, RT-qPCR, and western blotting analysis, we revealed that *PLA2G6* was markedly up-regulated in CMM tissues compared to nevus tissues, as well as remarkably increased *in vitro* in SK-MEL-28 and M14 melanoma cell lines compared to human melanocytes*. In vivo*, *PLA2G6* was also elevated in nine melanoma tissues compared to adjacent tissues. To investigate the malignant behaviors of PLA2G6 in CMM, SK-MEL-28 and M14 cell lines with *PLA2G6* stable knockdown by RNAi strategy were constructed. Through CCK8 and colony formation assays *in vitro* and xenograft tumor experiment *in vivo*, we found that knockdown of *PLA2G6* dramatically inhibited cell proliferation. The results of scratch-wound and transwell assays suggested that the migration and invasion of melanoma cells were prominently suppressed after silencing *PLA2G6*. In addition, flow cytometry showed that the knockdown of *PLA2G6* promoted the apoptosis rate of melanoma cells. To further explore the potential molecular mechanism, we used liquid chromatography coupled to tandem mass spectrometry (LC-MS/MS) proteomic and bioinformatics analysis. The GO and KEGG analysis suggested that the underlying mechanism of *PLA2G6* in CMM might be associated with the ferroptosis pathway, and ferroptosis-related proteins were validated to be differentially expressed in *PLA2G6* knockdown SK-MEL-28 and M14 cells. Together, these results suggested that *PLA2G6* knockdown significantly inhibited cell proliferation, metastasis, and promoted apoptosis in melanoma. Our findings on the biological function of *PLA2G6* and the underlying association between *PLA2G6* and ferroptosis in melanoma may contribute to developing a potential therapeutic strategy for melanoma.

## Introduction

Cutaneous malignant melanoma (CMM) remains the deadliest form of human cutaneous carcinoma, with markedly increasing incidence worldwide. CMM exhibits a high invasive behavior and tends to metastasize which poorly responds to conventional chemotherapy and radiotherapy, leading to poor prognosis (1). Therefore, comprehensive treatments like immunotherapies and molecularly targeted therapies should be applied to the treatments of advanced CMM (2). However, these regimens have received a little benefit (3). Hence, exploring the precise molecular mechanism is pivotal to the treatments of CMM and has attracted increasing attention.

The gene *PLA2G6* encodes a 85/88 kDa calcium-independent phospholipase A2 (iPLA_2_β) protein that can hydrolyze the sn-2 substituent of glycerophospholipids to release a lysophospholipid and a free fatty acid. It has been reported to participate in multiple biological processes, including cell proliferation, membrane remodeling, signal transduction, and apoptosis (4). The above functions of iPLA_2_β led to its activation in diseases such as neurological disorders, cancer, inflammatory, cardiovascular abnormalities and autoimmune disorders, and other metabolic disorders. In recent years, the role that iPLA_2_β plays in tumorigenesis has been relatively well studied (5). Several studies illustrated that *PLA2G6* was involved in proliferation, metastasis, and apoptosis in tumor cells (6–11). Pérez et al. demonstrated that the U937 human histiocytic lymphoma line express high levels of iPLA_2_β (6). Several other studies showed that iPLA_2_β promotes cancer cell growth *via* signal transduction pathways involving EGFR, MAPKs, p53, and p21 (7, 8). McHowat et al. reported the role of iPLA_2_β in tumor metastasis (9). They observed an 11-fold greater number of breast cancer cells in the lungs of wild-type mice compared to iPLA_2_β knockout mice, which suggested that iPLA_2_β may be responsible for cancer cell migration to distal locations. IPLA_2_β was also shown to promote the migration of hepatic cancer cells (10). In addition, other studies confirmed that iPLA_2_β is involved in the apoptosis of tumor cells. Nicotera et al. found that when prostate cancer cells were treated with bromoenol lactone, an iPLA_2_β inhibitor, the apoptosis rate of prostate cancer cells was increased (11).

Moreover, *PLA2G6* has also been linked to melanoma in the literature. Through genome-wide association studies (GWAS), single nucleotide polymorphisms (SNPs) in the loci *PLA2G6* were associated with nevus counts and melanoma risk, and *PLA2G6* was considered as a low penetrance risk gene for melanoma (12, 13). It is well known that a high nevus count is a critical established phenotypic marker of melanoma risk and is determined by underlying genetic factors and external factors (14). Although literature exists linking *PLA2G6* to melanoma, the expression level of *PLA2G6* and its biological role in melanoma is still unclear. The goal of the current study was to investigate the role of *PLA2G6* in the oncogenic progression of CMM and try to dissect the possible tumorigenic mechanism through an advanced proteomics strategy.

## Materials and Methods

### Cell Culture

CMM cell lines SK-MEL-28, M14, A375, and A875 were provided by the central laboratory of Institute of Dermatology, Chinese Academy of Medical Sciences & Peking Union Medical College and cultured in Dulbecco’s Modified Eagle’s medium (Gibco, USA) with 10% FBS (Gibco; USA) under 37°C and 5% CO_2_. Human epidermal melanocytes (MC) were separated from healthy donors’ prepuce. Prepuce was cut into strips and digested with 0.25% trypsin at 4°C overnight. The next day, the epidermis was separated from the dermis with fine forceps, incubated with 0.25% trypsin for 10 min, and neutralized with 10% FBS in DMEM. The collection was changed to suspension by repeated pipetting, filtered (100 μm filter), centrifuged at 1200 rpm for 4 min. The isolated melanocytes were resuspended in MelM media (ScienCell, USA) containing melanocyte growth supplement (ScienCell, USA) and penicillin/streptomycin solution (ScienCell, USA) under 37°C and 5% CO2.

### Lentivirus Transfection

According to the accession number NM_003560.4 of the human *PLA2G6* gene, we designed three interference sequences using short hairpin RNA (shRNA) design tools that were freely available at https://rnaidesigner.thermofisher.com/rnaiexpress/. The three shRNA sequences are as follows: 1. GCTGACGCCCTAGTGAATTTC; 2. GCAATGCTCGGTGCAACATCA; 3. GCATCCAGTACTTCAGATTGA. The negative control sequence (5’-GTTCTCCGAACGTGTCACGT-3’) was provided by GenePharma Technology Co., Ltd., with no homology to any human gene. The synthetic nucleotide acid fragments described above were annealed to form a double-stranded oligo, then inserted into the BamHI and EcoRI site restriction enzymes cleaved LV3-pGLV-h1-GFP-puro vector by homologous recombination using the T4 DNA ligase enzyme. Competent Top10 cells were prepared with calcium chloride and subsequently transformed. The recombinant plasmid was transformed into the competent cells, and the positive clones were screened by ampicillin medium. The plasmid was obtained and identified by alkali lysis method and gel electrophoresis. The *PLA2G6*-shRNA recombinant and assistant packaging plasmids were co-transfected into HEK293T cells (GenePharma, China). The lentiviral supernatant was concentrated, purified, and stored at −80°C for transfection. SK-MEL-28 and M14 cells were seeded in 6-well plates at a density of 1×10^5^ cells/well and infected with lentivirus of 20 multiplicity of infection (MOI) for 16 h. The culture medium was refreshed after that. GFP fluorescence expression was detected by fluorescence microscopy for infecting efficiency evaluation 72 h after infection. At the same time, 2 µg/mL puromycin (Solarbio, China) was used to screen the stable knockdown cells for more than 72 h continuously. The expression of target genes was detected by RT-qPCR or western blotting.

### Data Analysis of Public Databases

The mRNA expression levels of *PLA2G6* in various kinds of cancer were analyzed using CCLE (https://portals.broadinstitute.org/ccle/home), an online database of gene expression from 1,000 human cancer cell lines. The Talantov melanoma dataset obtained from the Oncomine database, including mRNA expression of 45 cutaneous melanoma samples and 18 benign skin nevus samples, was used to investigate the expression of *PLA2G6* in cutaneous melanoma.

### Clinical Patient Specimens

All tissue samples used in this research were provided by 61 melanoma patients (included 52 paraffin-embedded and 9 frozen tissue samples) and 27 benign melanocyte nevus patients who had undergone surgery at the Institute of Dermatology, Chinese Academy of Medical Sciences, and Peking Union Medical College (Nanjing, China). All samples were pathologically examined. The obtained clinical samples were stored at −80°C or embedded in paraffin at room temperature.

### Immunohistochemistry

A total of 52 cases of melanoma tissues and 27 nevi tissues were used for PLA2G6 immunohistochemical staining. After deparaffinization, hydration, and blocking, the five micrometer thick sections were incubated with the primary anti-PLA2G6 and anti-Ki-67 polyclonal antibody (1:1000; abcam, USA) overnight at 4°C and incubated with HRP-conjugated secondary antibody (DAKO, USA) for 30 min at room temperature. Staining used DAB Horseradish Peroxidase Color Development Kit (Beyotime, China). Two pathologists scored all sections under a light microscope and recorded. The immunohistochemical score was calculated by multiplying the positive cell score by the staining intensity score. The staining intensity scores were as follows: 0: no staining; 1: slightly yellow; 2: yellow-brown; 3: Brown. The positive cells were scored as follows: 0: 0–5%; 1:6–25%; 2:26–50%; 3:51–75%; 4: >75%. The final score was assigned four grades: score 0, negative (−); score 1–4, weak positive (+); score 5–8, middle positive (++); score 9–12, strong positive (+++).

### Reverse Transcription-Quantitative PCR (RT-qPCR)

Total RNA was extracted by Trizol reagent (Invitrogen, USA), and the cDNA was synthesized with PrimeScript™ RT Master Mix (Toyobo Life Science, Japan). In brief, 1 μg total RNA was used to reverse transcribe 20 μL cDNA, and 1.5 μL of cDNA was used for each qPCR. RT-qPCR experiments were performed using the SYBR-Green Premix Ex Taq kit (Takara, Japan) and were assessed by Roche Lightcycler 480 Real-Time PCR System. The following cycling conditions were used for qPCR: preheat for 1 cycle at 94°C for 30 sec, amplification for 40 cycles at 94°C for 5 s, 60°C for 10 s and cooling to 50°C for 30 s. The primers were listed as follows: *PLA2G6*, forward primer: 5’-TTTGGCCGCCTGGTCAATAC-3’ and reverse primer: 5’-CTCCCGAACTCGGTCACTC-3’; *GAPDH*, forward primer: 5’-CATCTTCTTTTGCGTCGCCA-3’ and reverse primer: 5’-TTAAAAGCAGCCCTGGTGACC-3’. The relative expressions of mRNAs were evaluated by the 2^−ΔΔCq^ method. Three independent experiments were performed.

### Western Blotting

Total protein was extracted using RIPA lysis buffer (Beyotime, China), and protein concentrations were detected using a BCA kit (Beyotime, China). Protein samples were separated by 4-20% SDS-PAGE gel and transferred onto the PVDF membrane (Millipore, USA). The membrane was then blocked in TBST buffer containing 5% bovine serum albumin. After incubation at 4°C overnight with primary antibodies, the blot was probed with HRP-conjugated anti-mouse/rabbit secondary antibodies (1:2000; Cell Signaling Technology, USA) at room temperature for 1 h. Then the immunoreactive bands were detected using an enhanced chemiluminescence kit (Thermo Scientific, USA). The primary antibodies included PLA2G6 (1:200, Santa Cruz Biotechnology, USA), CP (1:1000, Abcam, USA), TF (1:1000, Abcam, USA), FTL (1:5000, Abcam, USA), PTGS2 (1:1000, ProteinTech, China), GPX4 (1:1000, ProteinTech, China), SLC7A11 (1:1000, ProteinTech, China) and GAPDH (1:2000; Abcam, USA). Three independent experiments were performed.

### Cell Proliferation, Migration, Invasion, and Apoptosis Assay *In Vitro*

CCK-8 and colony formation assay were used to detect cell proliferation *in vitro*. SK-MEL-28 and M14 cells in the logarithmic growth phase were seeded in 96-well plates (2,500 cells/well) for 24 h. The cells were incubated in a medium with 10% CCK8 reagent (Dojindo Molecular Technologies, Inc., Japan) at 37°C for 1 h. The optical density (OD) absorption value at 450 nm was recorded at 24, 48, and 72 h, respectively. Next, cells were seeded in a 6-well plate at 1000 cells/well for colony formation assay. The medium was changed every 4 days. After macroscopic colonies could be observed, cells were fixed in 4% paraformaldehyde and stained with 0.1% crystal violet for 15 min and counted.

Cell migration and invasion ability were detected by scratch-wound assay and transwell assay. SK-MEL-28 and M14 cells were seeded into a 6-well plate until 90% confluency was reached. Then, the vertical scratch-wound was scraped with a 10 μL sterile suction head. Cells were cultured with a serum-free medium after removing the cell debris. The relative distance between two edges was measured by photos taken at 0 h and 24 h.

For transwell assay, cells were seeded in the upper chambers of Matrigel (BD Biosciences, USA) -uncoated or -coated transwell inserts (Corning Costar, USA) at a density of 2×10^4^ cells/well. The upper chambers were cultured by serum-free medium, and the lower chambers was added into medium with 10% FBS. After 24 h, the migrating or invading cells were fixed with 4% paraformaldehyde and stained with 0.1% crystal violet for 15 min, respectively.

For apoptosis assay, cells were seeded in 6-well plates until 90% confluency was reached. Then cells were collected and resuspended in binding buffer at a concentration of 1×10^6^ cells/mL. A total of 5 μL Annexin V-PE and 5 μL 7-AAD were mixed and reacted for 15 min in the dark at room temperature. The apoptotic cells were analyzed by FACSVerse™ (Becton, Dickinson, and Company) with BD FACSuite™ software. Three independent experiments were performed.

### Animal Experiments

A total of 10 female BALB/c nude mice (4-6 weeks old) were supplied by Shanghai SLAC Laboratory Animal Co., Ltd (Shanghai, China). The nude mice were randomly divided into different treatment groups with five mice each, and maintained in a pathogen-free animal facility, followed by subcutaneously injection with 1×10^7^ cells/mL M14 cells in 100 μL PBS on the right side to establish a subcutaneous xenograft model. The tumor volume was measured with calipers every 4 days and was calculated with the formula volume = 0.5×(length×width^2^). The mice were euthanized after 4 weeks before the tumor reached 2000 mm^3^. Tumor tissues were separated, weighed, photographed, and embedded in paraffin for IHC analysis.

### Protein Extraction and Liquid Chromatography-Tandem Mass Spectrometry (LC-MS/MS Analysis)

The stable knockdown M14 cells were lysed in SDT buffer (4%SDS, 100 mM Tris-HCl, 1 mM DTT, pH7.6), and protein concentrations were determined by a BCA kit (Bio-Rad, USA). Protein was digested by trypsin and was proceeded according to the filter-aided sample preparation (FASP) protocol described by Matthias Mann (15). TMT reagent was used for each sample to label 100 μg peptide mixture of each sample according to the manufacturer’s instructions (Thermo Fisher Scientific, Inc.). Labeled peptides were fractionated by High pH Reversed-Phase Peptide Fractionation Kit (Thermo Fisher Scientific, Inc.). The collected fractions were desalted on C18 Cartridges (Empore™ SPE Cartridges C18, bed I.D. 7 mm, volume 3 mL, Sigma) and concentrated by vacuum centrifugation until analysis by mass spectrometry. The HPLC liquid phase system Easy-nLC was used for separation. The peptides were loaded onto a reverse-phase trap column (Thermo Scientific Acclaim PepMap100, 100 μm×2 cm, nanoViper C18) connected to the C18-reversed-phase analytical column (Thermo Scientific Easy Column, 10 cm long, 75 μm inner diameter, 3 μm resin) in buffer A (0.1% Formic acid) and separated with a linear gradient of buffer B (84% acetonitrile and 0.1% Formic acid) at a flow rate of 300 nL/min controlled by IntelliFlow technology. After chromatographic separation, the peptides were analyzed by Q-Exactive Plus mass spectrometer (Thermo Scientific, Waltham, USA) with the following parameters: analysis duration, 90 min; detection method, positive ion; scanning range of parent ion, 300–1800 *m/z*. The resolution of the first-level mass spectrometry was 70,000 at 200 m/z. The maximum injects time to 50 ms, and the dynamic exclusion duration was 60 s. Mass charge ratios of polypeptides and polypeptide fragments were collected as follows: 20 fragment profiles (MS2 scan) were collected after each full scan. The MS2 activation type was HCD. The isolation window was 2 *m/z*. The normalized collision energy was 30 eV and the underfill ratio was defined as 0.1%.

### Bioinformatic Analysis

The protein with *P*-value < 0.05 and fold change > 1.2 was considered the differentially expressed protein (DEP). DEPs were subjected to bioinformatics annotation using the Gene Ontology (GO) annotation database (http://www.geneontology.org) and Kyoto Encyclopedia of Genes and Genomes (KEGG) database (http://www.genome.jp/kegg/) to identify the functions or interactions between these DEPs.

### Statistical Analysis

All experiments in this research were performed at least three times, and quantitative data were expressed as the mean ± standard error. All data analysis except CCLE in this article was conducted using GraphPad Prism 8.0 Software (GraphPad, Inc., San Diego, California, USA). CCLE data was conducted using R software. In *in vitro* and *in vivo* experiments, the differences between the two groups were evaluated using Student’s t-test. In contrast, three or more groups were determined using a one-way ANOVA test. *P* < 0.05 was considered statistically different.

## Results

### PLA2G6 Expression Is Elevated in Cutaneous Melanoma

**Figure 1:**
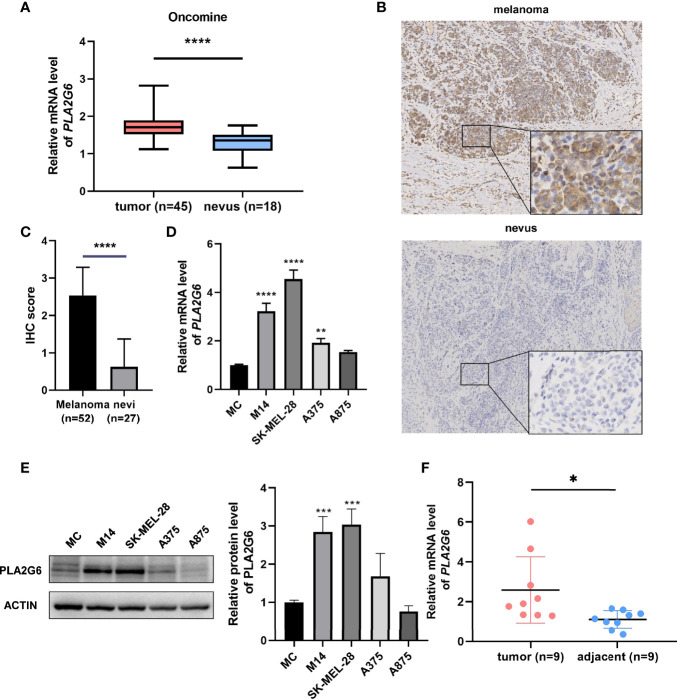
PLA2G6 expression was elevated in cutaneous melanoma. **(A)** The expression file of Oncomine showed different mRNA levels of *PLA2G6* in normal nevi samples (n = 18) and melanoma samples (n = 45). **(B)** Immunohistochemistry staining for PLA2G6 in clinical melanoma tissue and nevus tissue (the large panel was at a 100× magnification and the small panel was at a 400× magnification). **(C)** The immunostaining scores of melanoma tissues (n = 52) and nevus tissues (n = 27) were displayed using t test. **(D)** The mRNA level and **(E)** protein level of PLA2G6 in normal human epidermal melanocytes (MC) and four typical melanoma cell lines M14, SK-MEL-28, A375, and A875. **(F)** The mRNA level of *PLA2G6* was detected by RT-qPCR between melanoma tissues (n = 9) and adjacent non-tumor tissues (n = 9). *P < 0.05, **P < 0.01, ***P < 0.001, ****P < 0.0001.

**Figure 2:**
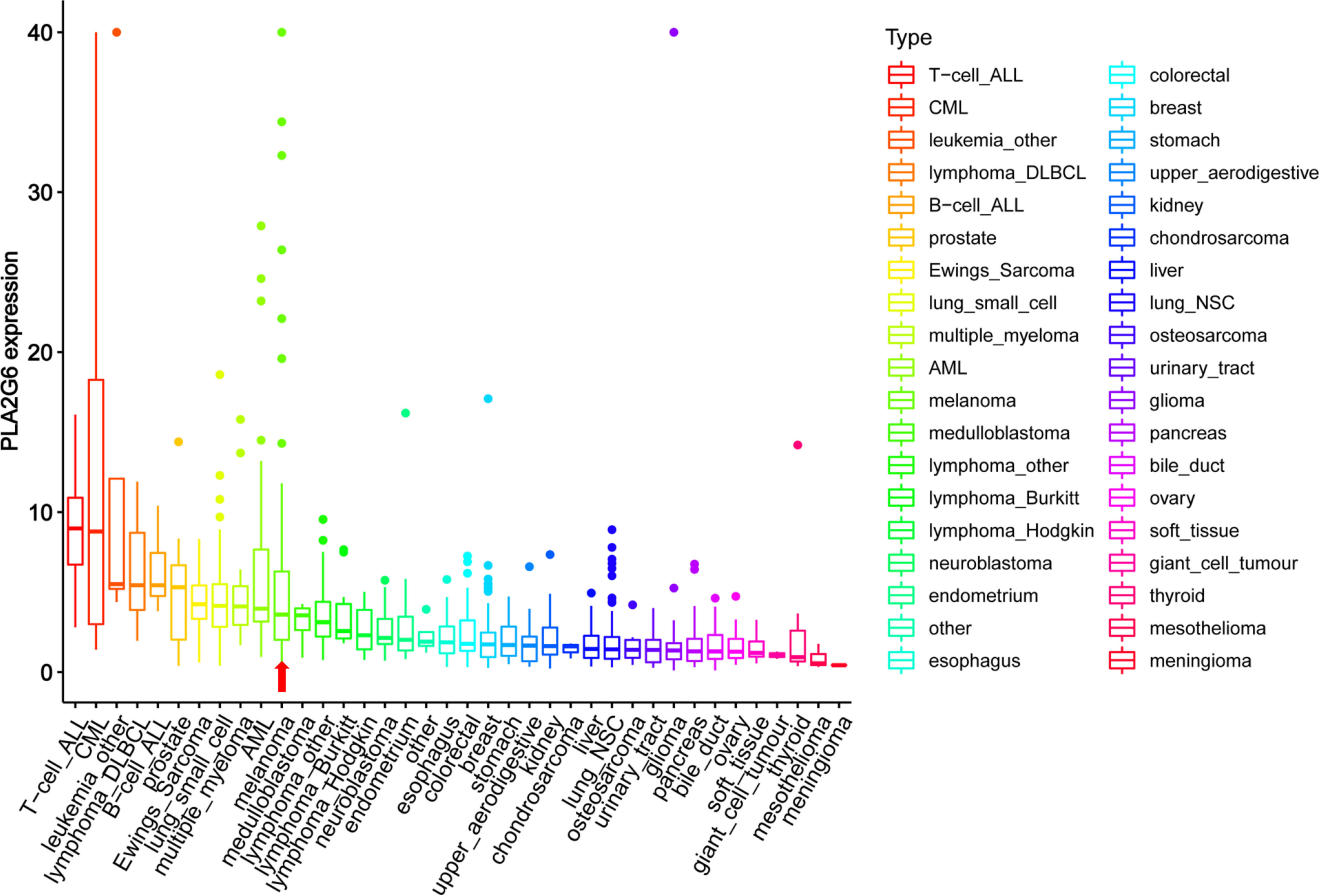
The mRNA levels of *PLA2G6* in various cancer cell lines were obtained at Cancer Cell Line Encyclopedia (CCLE). The red arrow showed the mRNA levels of *PLA2G6* in melanoma.

Based on the Oncomine database, we found that the mRNA level of *PLA2G6* was up-regulated in CMM compared with the benign melanocytic nevus (**Figure 1A**). Next, the CCLE data indicates that the expression of *PLA2G6* was increased in melanoma cell lines (**Figure 2**), which is consistent with the Oncomine analysis. Furthermore, immunohistochemical results showed that PLA2G6 protein was mainly located in the cell cytoplasm. CMM tissue had a higher level and nevus tissue had a lower level of PLA2G6 expression (**Figures 1B, C**). To characterize the PLA2G6 activity *in vitro*, we further examined the mRNA and protein expression level of PLA2G6 in normal human epidermal MC and four typical melanoma cell lines M14, SK-MEL-28, A375, and A875. *PLA2G6* expression was remarkably higher in M14 and SK-MEL-28 cell lines than in MC by RT-qPCR analysis (**Figure 1D**) and western blotting analysis (**Figure 1E**). Hence, we used those two cell lines as models to assess the biological effects of PLA2G6 on melanoma. Moreover, nine pairs of melanoma and adjacent non-tumor tissues were detected by RT-qPCR analysis. The results showed that the mRNA level of *PLA2G6* was notably up-regulated in melanoma tissues (**Figure 1F**).

### *PLA2G6* Knockdown Inhibits CMM Cells Proliferation, Metastasis and Promotes Apoptosis *In Vitro*

**Figure 3:**
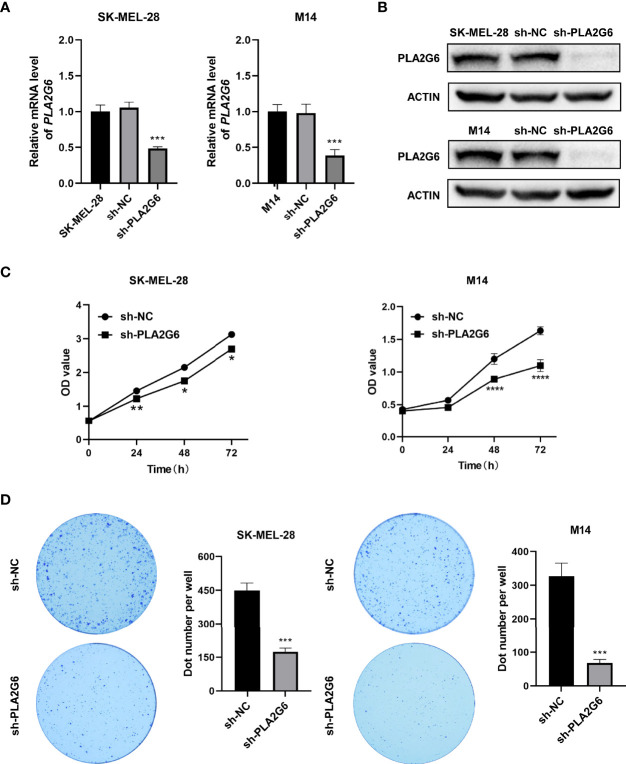
*PLA2G6* knockdown suppresses the proliferative ability of SK-MEL-28 and M14 melanoma cells *in vitro*. **(A)** RT-qPCR and **(B)** Western blotting analysis was performed to detect the knockdown efficiency of sh-PLA2G6 and sh-NC. **(C)** Cell viability was measured by CCK-8 assay in SK-MEL-28 and M14 cells. **(D)** Images of colony formation and the quantification of the number of colonies are exhibited. *P< 0.05, **P < 0.01, ***P < 0.001, ****P < 0.0001.

To verify the biological function of *PLA2G6* in melanoma cells, both SK-MEL-28 and M14 cell lines with *PLA2G6* stable knockdown by RNAi strategy were constructed. The knockdown efficiency of the most effective *PLA2G6*-targeted shRNA sequence (5’-GCATCCAGTACTTCAGATTGA -3’) in SK-MEL-28 and M14 cells were examined by RT-qPCR (**Figure 3A**) and western blotting (**Figure 3B**), respectively. The results showed that PLA2G6 expression was successfully and significantly reduced in SK-MEL-28 and M14 cells.

The proliferation of melanoma cells was assessed by CCK8 and colony formation assay. The data of CCK8 assay showed that the OD values were dramatically lower in the *PLA2G6* knockdown groups than those in the control groups at 48 and 72 h time points (**Figure 3C**). And colony formation assays consistently indicated that *PLA2G6* knockdown reduced the colonies formed by SK-MEL-28 and M14 cells, especially by M14 (**Figure 3D**). Those results suggested that *PLA2G6* knockdown reduced the growth of SK-MEL-28 and M14 cells.

**Figure 4:**
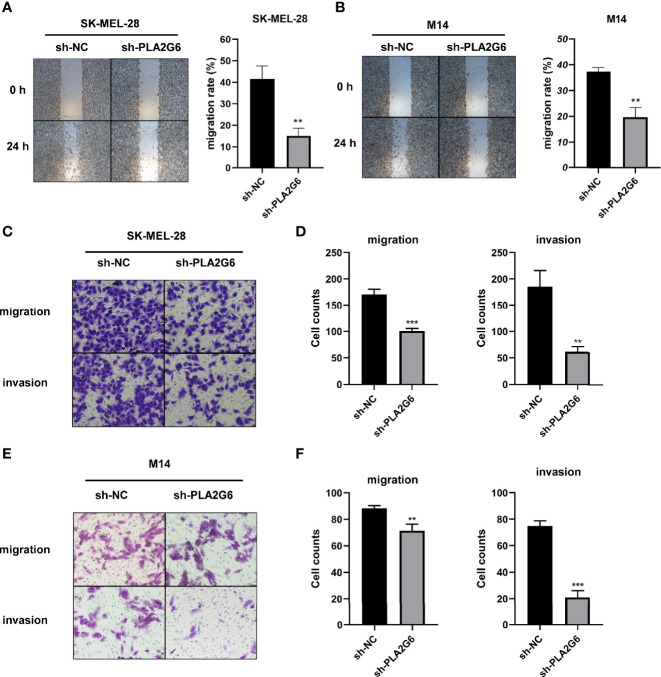
*PLA2G6* knockdown inhibits migration and invasion of CMM cells. **(A)** Representative microscopic images and quantification of scratch wound assays in SK-MEL-28 and **(B)** M14 cells. **(C)** The representative images (magnification × 200) and number of migrated cells and **(D)** invaded cells of SK-MEL-28. **(E)** The representative images (magnification ×200) and number of migrated cells and **(F)** invaded cells of M14. **P < 0.01, ***P < 0.001.

The effect of *PLA2G6* on melanoma metastasis was investigated by scratch wound assay and transwell assay. After 24 h of scratching, we found that the migration distances of SK-MEL-28 and M14 cells silenced *PLA2G6* were shorter than those of the control groups (**Figures 4A, B**). Transwell assays further confirmed the results obtained in scratching assay. The numbers of migrated and invaded cells in the sh-PLA2G6 groups were dramatically less than those in the sh-NC group in SK-MEL-28 cells (**Figures 4C, D**) and M14 cells (**Figures 4E, F**). In conclusion, silencing *PLA2G6* markedly impaired the migration and invasion behavior of melanoma cells.

**Figure 5:**
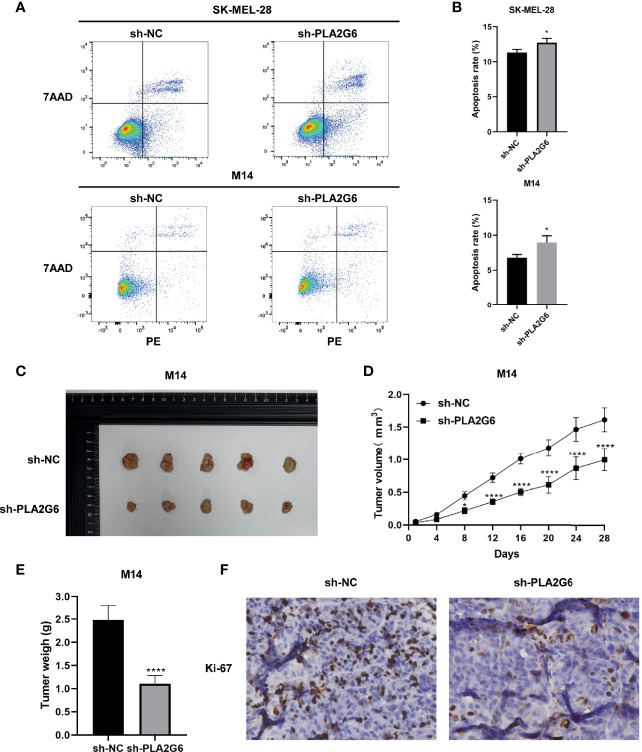
*PLA2G6* knockdown promotes apoptosis *in vitro* and suppressed xenograft tumor growth in nude mice. **(A, B)** Cell apoptosis of SK-MEL-28 and M14 cells detected by flow cytometry. **(C)** Representative images of xenograft tumor mass sacrificed at 4 weeks. **(D)** Tumor volume was assessed every 4 days. **(E)** Average tumor weight was determined after the mice were sacrificed at the end of treatment. **(F)** Proliferation marker Ki-67 was detected by immunohistochemical staining. *P < 0.05, ****P < 0.0001.

Annexin V-PE/7-AAD double staining combined with flow cytometry was employed to examine the apoptoses of SK-MEL-28 and M14 cells. The data showed that in SK-MEL-28 and M14 cells, the apoptotic rates of *PLA2G6* depletion groups were higher than those of control groups to some extent (**Figures 5A, B**). Taken together, *PLA2G6* knockdown inhibits CMM cells proliferation, metastasis and promotes apoptosis *in vitro*.

### *PLA2G6* Knockdown Suppresses the Growth of Tumor Xenograft in Nude Mice

We further detected the effect of *PLA2G6* knockdown on tumor xenograft growth in the BALB/c nude mice injected with M14 cells. The tumor growth of *PLA2G6* deficiency group was slower than that of control group (**Figures 5C, D**). By the end of the experiment, the tumor weight of *PLA2G6* deficiency group was notably reduced (**Figure 5E**). As evidence, the proliferation marker Ki-67 in *PLA2G6* knockdown tumors was significantly down-regulated, illustrated by immunohistochemistry staining (**Figure 5F**). These results suggested that the knockdown of *PLA2G6* suppresses the growth of tumors *in vivo*.

### Dissecting the Possible Regulatory Mechanism of *PLA2G6* in CMM by Quantitative Proteomics

**Figure 6:**
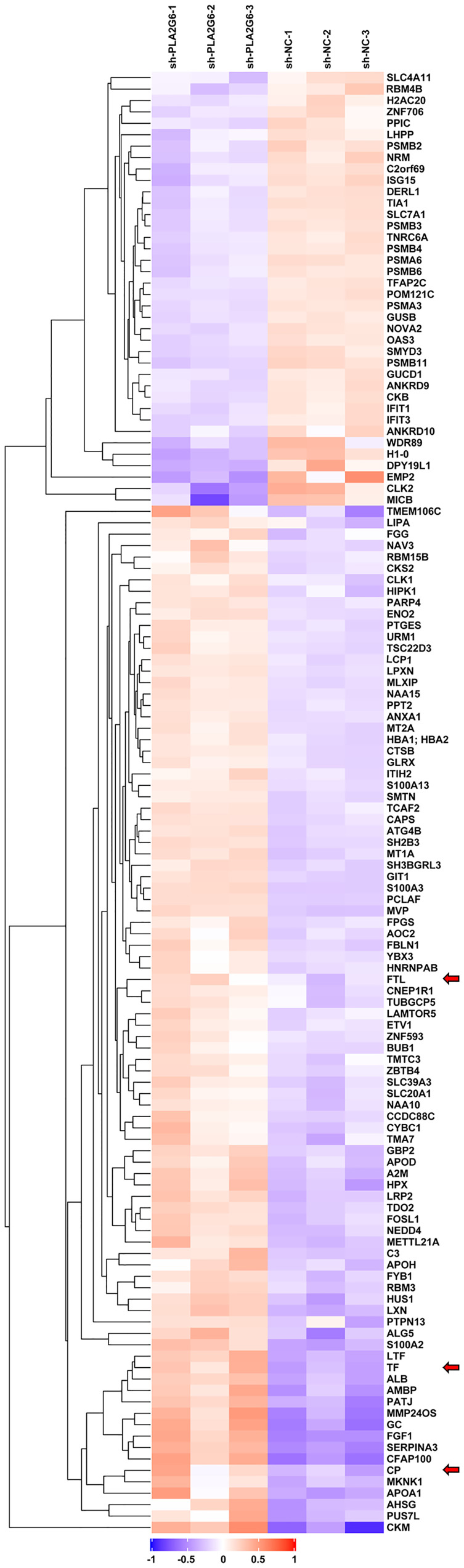
The heat map displays protein expression changes in the 3 *PLA2G6* knockdown-treated and 3 control samples. Red, white, and blue represent increased, unchanged, and decreases expression, respectively. Three ferroptosis-related DEPs were indicated by red arrows.

To explore the possible molecular mechanism of action of *PLA2G6* in melanoma, we used TMT quantitative proteomics technology to compare the whole-cell proteomes in control and *PLA2G6* knockdown M14 cells. As a result, 114367 spectra out of 564320 total spectra were matched after quality control. A total of 52652 unique peptides were identified, and 7281 proteins were obtained. A total of 128 DEPs were obtained, while 90 proteins were up-regulated and 38 were down-regulated. These proteins were well distinguished in the heatmap (**Figure 6**).

**Figure 7:**
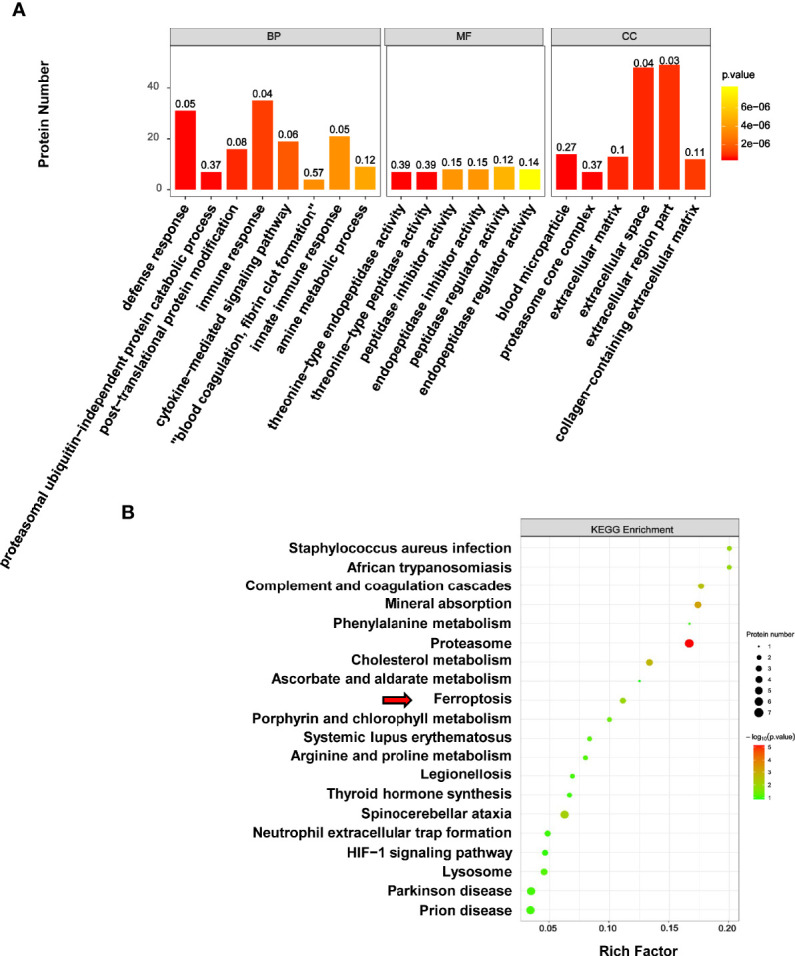
GO and KEGG analysis of differentially expressed proteins associated with melanoma. **(A)** The results of GO reassignments showed that proteins were categorized biological process (BP), molecular function (MF), and cellular component (CC). **(B)** The top enriched 20 KEGG pathways were shown in scatter plot, and the ferroptosis pathway was indicated by red arrow.

The results of GO reassignments showed that proteins were categorized into three ontologies: Biological Process (BP), Molecular Function (MF), and Cellular Component (CC). The most enriched GO terms of BP, MF and CC were annotated as immune response, peptidase regulator activity and extracellular region part, respectively (**Figure 7A**).

To explore how PLA2G6 plays a role in melanoma by these cellular processes, DEPs were then mapped to the reference pathways in the KEGG database. The bubble graph was used to show the top 20 enriched KEGG pathways (**Figure 7B**). Interestingly, only the ferroptosis pathway was the most relevant Biological Process to the tumor and three genes were mapped to the ferroptosis pathway, which included transferrin (TF), ceruloplasmin (CP), and ferritin light chain (FTL). All these 3 genes were up-regulated in *PLA2G6* knockdown cells.

### Validation of the Expression of Ferroptosis-Related Proteins in Response to *PLA2G6* Knockdown

**Figure 8:**
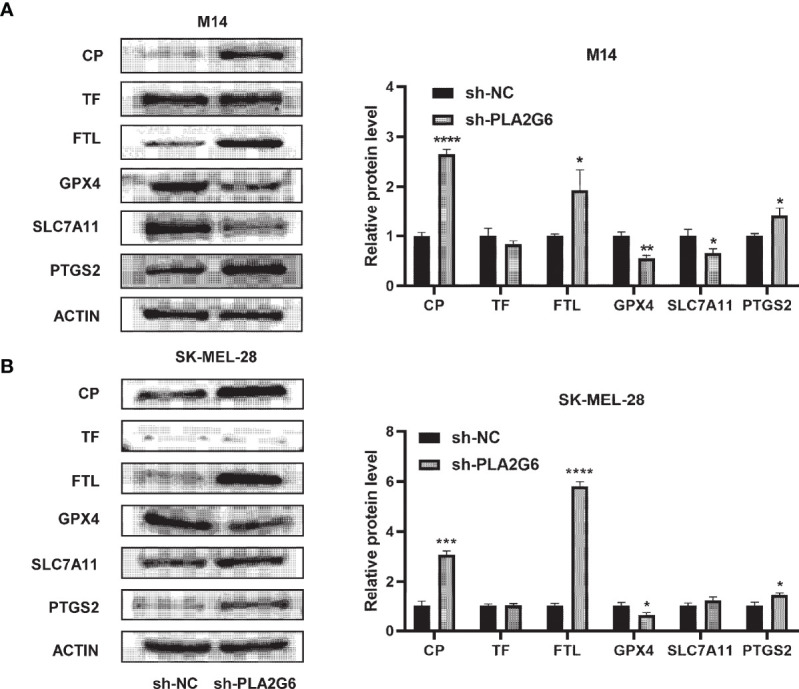
Validation of the expression of ferroptosis-related proteins by western blotting in *PLA2G6* knockdown SK-MEL-28 **(A)** and M14 cells **(B)**. *P < 0.05, **P < 0.01, ***P < 0.001, ****P < 0.0001.

Western blotting analysis was used to further confirm the differential expression levels of ferroptosis-related proteins identified by the proteomics technique. The results showed that the protein expression levels of CP and FTL were significantly up-regulated in *PLA2G6* knockdown M14 cells and SK-MEL-28 cells, which were in high accordance with proteomic assay results. However, there was no significant difference in TF protein expression. Moreover, we also detected the protein expression levels of ferroptosis biomarkers, including GPX4, SLC7A11, and PTGS2. Reduced expression of GPX4 and elevated expression of PTGS2 were observed in both M14 cells and SK-MEL-28 cells, but reduced expression of SLC7A11 was only detected in M14 cells (**Figures 8A, B**).

## Discussion

In this study, we detected the functions of *PLA2G6* in the progression of melanoma and demonstrated that *PLA2G6* played an oncogenic role in melanoma by up-regulating cell proliferation, migration, invasion and down-regulating cell apoptosis. Moreover, quantitative proteomics analysis further reveals that *PLA2G6* might achieve the above functions by affecting the ferroptosis pathway.

Encoded by *PLA2G6*, iPLA_2_β is a member of the phospholipases A2 family that hydrolyze the sn-2 substituent from membrane phospholipids characterized by not requiring Ca^2+^ for catalytic activity and translocation to the membrane. *PLA2G6* has a vital role in many physiological processes. Several studies have demonstrated that *PLA2G6* is closely related to many tumors as an oncogene. The expression of *PLA2G6* from the online databases found that *PLA2G6* is overexpressed in many human cancers (**Figure 2**). As mentioned above, *PLA2G6* was involved in multiple malignant behaviors in tumor cells, including proliferation, metastasis, and apoptosis (6–11). However, to the best of our knowledge, the expression and function of *PLA2G6* have never been explored in human CMM.

Our study, based on Oncomine expression analysis, mRNA and protein level of CMM tissues and cell lines, confirmed that *PLA2G6* is severely up-regulated in CMM tissues compared with normal tissues and skin nevi. Our study successfully and significantly down-regulated the expression of *PLA2G6* in SK-MEL-28 and M14 cell lines. Through CCK8 assay, cell clonal formation, and xenograft model of nude mice, we found that the proliferation of melanoma cells was significantly attenuated after *PLA2G6* knockdown, suggesting that *PLA2G6* can promote the proliferation of melanoma cells *in vitro* and *in vivo*. Scratch wound and transwell assays showed that knockdown of *PLA2G6* reduced migration and invasion in melanoma cells. Moreover, Annexin V-PE/7-AAD staining assay revealed that depletion of *PLA2G6* increased the apoptotic rate in SK-MEL-28 and M14 cells to some extent. Thus, our data strongly suggested that *PLA2G6* acted as an oncogene in CMM.

To further explore the mechanism of *PLA2G6* action in CMM, proteomics analysis and subsequent validation tests were performed. The results showed that the reduction of iPLA2β affects ferroptosis-related proteins, including iron transport-related proteins CP, FTL and GSH metabolic-related proteins GPX4, SLC7A11. Ferroptosis is a caspase-independent cell death modality characterized by the overwhelming production of reactive oxygen species (ROS) and the accumulation of iron-dependent lipid peroxides (16). Although previous research described the relationship between ferroptosis and melanoma (17), no finding has been reported whether *PLA2G6* is involved in this process in melanoma. In our study, KEGG analysis shows that *PLA2G6* knockdown was associated with ferroptosis process in melanoma. Similarly, in a few recent articles*, PLA2G6* encoded protein, iPLA_2_β, has been closely associated with ferroptosis (18–20). Based on the ability of iPLA_2_β can hydrolyze the membrane phospholipids and release free fatty acid, previous studies usually investigated the relationship between iPLA_2_β and ferroptosis through a metabolic pathway. Chen et al. found that iPLA_2_β was a direct target of p53 and controlled p53-driven ferroptosis in cancer cells by cleaving peroxidized lipids for detoxification (18). Moreover, Sun et al. demonstrate that the loss function of iPLA_2_β leads to sensitivity to ferroptosis in dopamine neurons and iPLA_2_β can act as an anti-ferroptotic guardian *via* elimination of the pro-ferroptotic signal, 15-HpETE-PE (19). In addition, Beharier et al. proved that PLA2G6-deficient trophoblasts exhibit enhanced sensitivity to ferroptosis when GPX4 activity is absent. However, at baseline and in the presence of intact GPX4, deletion of *PLA2G6* was not sufficient to induce ferroptosis in Placental Trophoblasts (20). This study observed that caspase activation was relatively mild when *PLA2G6* was depleted. Therefore, we speculated that *PLA2G6*, as an oncogene, may synergistically regulate cell death through ferroptosis and apoptosis pathways. Furthermore, the significantly increased protein level of CP and FTL suggested that iPLA_2_β may influence ferroptosis mainly through the pathway of iron ion transport process rather than the lipids detoxification function mentioned above.

In addition, the correlation between iPLA_2_β and iron transport was reported in other studies. In neurodegeneration with brain iron accumulation (NBIA), Goichi et al. found that marked iron deposition and peroxidized lipids were increased in the brains of iPLA_2_β-KO mice, along with degeneration of the mitochondrial inner membrane in iPLA_2_β-KO cells (21). In the same study, the authors verified that the *PLA2G6* knockdown could activate and up-regulate IRP2 and DMT1, leading to iron uptake. Recent studies demonstrate that CP plays a vital role in iron homeostasis. Fe^2+^ was transported out of the cell by ferroportin and was oxidized by CP to facilitate loading on TF (22). Besides, CP can also regulate ferroptosis. Shang et al. found that erastin- and RSL3-induced ferroptosis was promoted under the genetic ablation of CP accompanied by the accumulation of intracellular Fe^2+^ and lipid ROS, which suggested that CP can regulate iron homeostasis and suppress ferroptosis in hepatocellular carcinoma cells (23).

Together, we speculate that iPLA_2_β may suppress ferroptosis by regulating iron transport or stored related proteins, which lays a solid theoretical foundation for us to reveal its molecular mechanism in future studies deeply. Although the underlying mechanism of iPLA_2_β-mediated ferroptosis needs further elucidation, these data suggest that iPLA_2_β is involved in regulating ferroptosis in melanoma cells and may affect ferroptosis in a variety of ways.

## Conclusion

In summary, for the first time, our study identified the *PLA2G6* as an oncogene that promoted proliferation, metastasis, and inhibited apoptosis in CMM cells. In addition, we established the association between *PLA2G6* and ferroptosis in the melanoma cells through proteomics that may inspire *PLA2G6*-targeted therapeutic strategies.

## Data Availability Statement

The original contributions presented in the study are included in the article/**Supplementary Material**. Further inquiries can be directed to the corresponding authors.

## Ethics Statement

The studies involving human participants were reviewed and approved by Ethics Committee of the Institute of Dermatology, Chinese Academy of Medical Sciences, and Peking Union Medical College. The patients/participants provided their written informed consent to participate in this study. The animal study was reviewed and approved by Ethics Committee of the Institute of Dermatology, Chinese Academy of Medical Sciences, and Peking Union Medical College.

## Author Contributions

YFW, JQ, XX, and JS conceived and designed the experiments. YFW performed all the experiments and did data analysis. QM, HS, and YW provided technical support and discussed the results. YFW wrote the draft. JQ, XX, and JS provided critical comments, suggestions, and revised the manuscript. HS, YW, XX, and JS provided funding support. All authors contributed to the article and approved the submitted version.

## Funding

This work was supported by the National Natural Science Foundation of China (81772916, 82103470, 81872216) and the Jiangsu Natural Science Foundation (BK20171132).

## Conflict of Interest

The authors declare that the research was conducted in the absence of any commercial or financial relationships that could be construed as a potential conflict of interest.

## Publisher’s Note

All claims expressed in this article are solely those of the authors and do not necessarily represent those of their affiliated organizations, or those of the publisher, the editors and the reviewers. Any product that may be evaluated in this article, or claim that may be made by its manufacturer, is not guaranteed or endorsed by the publisher.
